# Identification of Epigenetic Interactions between miRNA and Gene Expression as Potential Prognostic Markers in Bladder Cancer

**DOI:** 10.3390/genes13091629

**Published:** 2022-09-10

**Authors:** Amira Awadalla, Hassan Abol-Enein, Eman T. Hamam, Asmaa E. Ahmed, Salma M. Khirallah, Ahmed El-Assmy, Sally Abdallah Mostafa, Ahmed O. Babalghith, Mohamed Ali, Mona Abdel-Rahim, Ahmed A. Shokeir, Ahmed M. Harraz

**Affiliations:** 1 Center of Excellence for Genome and Cancer Research, Urology and Nephrology Center, Mansoura University, Mansoura 35516, Egypt; 2 Biochemistry Division, Chemistry Department, Faculty of Science, Port Said University, Port Said 42526, Egypt; 3 Medical Biochemistry and Molecular Biology Department, Faculty of Medicine, Mansoura University, Mansoura 35516, Egypt; 4 Medical Genetics Department, College of Medicine, Umm Al-Qura University, Makkah, Saudi Arabia; 5 Biochemistry Division, Chemistry Department, Faculty of Science, Zagazig University, Zagazig 44519, Egypt

**Keywords:** bladder cancer, miRNAs, mRNA, protein expression, prognosis, Wnt/β-catenin

## Abstract

**Purpose:** To identify the role of a set of microRNAs and their target genes and protein expression levels in the pathogenesis of bladder cancer with a muscular invasion (T2–T4) and non-muscular invasion (T1). **Methods:** In 157 patients, bladder specimen was examined for the expression of a set of miRNAs including let-7a-5p, miRNA-449a-5p, miRNA-145-3P, miRNA-124-3P, miRNA-138-5p, and miRNA-23a-5p and their targeted genes; *β-catenin*, *WNT7A*, *IRS2*, *FZD4*, *SOS1*, *HDAC1*, *HDAC2*, *HIF1α*, and *PTEN* using the qRT-PCR technique. The prognostic effect of miRNAs and their targeted genes on cancer-specific survival (CSS) was evaluated in pT2–pT4 stages. **Results:** pT1 was found in 40 patients while pT2–4 was found in 117 patients. The expression of let-7a-5P, miR-124-3P, miR-449a-5P, and miR-138-5P significantly decreased in pT2–4 compared with pT1 (*p* < 0.001), in contrast, miR-23a-5P increased significantly in pT2–pT4 compared with pT1 (*p* < 0.001). Moreover, the expression of miR-145 did not show a significant change (*p* = 0.31). Higher expression levels of *WNT7A*, *β-catenin*, *IRS2*, *FZD4*, and *SOS1* genes were observed in pT2–pT4 compared with pT1, whereas *HDAC1*, *HDAC2*, *HIF1α*, and *PTEN* genes were downregulated in pT2–pT4 compared with pT1. Lower CSS was significantly associated with lower expression of let-7a-5P, miR-124-3P, miR-449a-5P, and miR-138-5P. Higher expression of *β-catenin*, *FZD4*, *IRS2*, *WNT7a*, and *SOS1* was significantly associated with worse CSS. In contrast, lower levels of *HDAC1*, *HDAC2*, *HIF1α*, and *PTEN* were associated with lower CSS. **Conclusion:** Our results support let-7a-5P, miR-124-3P, miR-138-5P, and their target genes can be developed as accurate biomarkers for prognosis in bladder cancer with a muscular invasion.

## 1. Introduction

Bladder cancer (BC) is the sixth most common cancer in both men and women worldwide, and the fourth in men. The most frequent histologic type of BC is urothelial carcinoma (UC), which accounts for 90% of all cases [1]. The most widespread type of urothelial carcinoma of the bladder (UCB) is urothelial carcinoma (TCC), which is split into two groups: non-muscle invasive (NMIBC) and muscle-invasive (MIBC) bladder cancer. Intravesical therapies and transurethral resection are effective treatments for NMIBC (pTa and pT1), whereas chemo-radiotherapy or radical cystectomy are effective treatments for MIBC (T2–T4) [2].

Molecular insights into BC might allow more details to understand this complex disease. Therefore, studying the signaling pathways altered in BC such as the cell cycle pathway, DNA repair pathway, chromatin remodeling, angiogenesis, and histone modifications might have a significant impact on the BC prognosis [3].

The Wnt/β-catenin pathway is required for the normal urothelium regeneration in the bladder following injury; therefore, its role in the development of bladder cancer appears reasonable. Furthermore, β-catenin and Frizzled Class Receptor 4 (FZD4) are overexpressed in human bladder cancer, and Wnt/β-catenin signaling is activated by FZD4 stimulation, contributing to tumor progression. The increase in β-catenin in the nucleus has also been linked to worse effects in bladder cancer patients [4]. Wnt-targeted genes were linked to tumor grade and stage, indicating that they could be used as epigenetic biomarkers. HIF-1α and HIF-2α interact with β-catenin but at different locations. HIF-2 was revealed to be essential for β-catenin activation and proliferation in RCC cells. The interaction of β-catenin and HIF-2α opposes that of β-catenin on HIF1-α, suggesting that the HIF-1α/HIF2α ratio could influence cell growth [4]. In cancer, PI3K/Phosphatase/tensin homolog (*PTEN*) and Wnt/β-catenin signaling pathways correlate with each other as Wnt/β-catenin and PI3K being overexpressed with the inactivity/loss of PTEN [5]. In addition, insulin receptor substrate 2 (*IRS2*), which is elevated in insulin signaling pathways through let-7a, has recently gained wide attention in cancer development and progression [6]. Other biomarkers include Son of sevenless homolog 1 (*SOS1*) that is stimulated by ERK signaling activation, especially in higher stages of cancer. In addition, histone deacetylases (*HADC1* and *HADC2*) inhibition increases apoptosis through p53 and p21cip1/WAF1 in cancer [7]. HDAC dysregulation plays a significant role in many human cancers. HDACs are regulated via a variety of methods, including posttranslational alterations. Reversible phosphorylation is a typical posttranslational modification of HDACs. Many HDAC phosphorylations have particular implications. Some of these alterations support disease development [8].

MicroRNAs (MiRNAs) are non-coding RNA sequences with a length of 19–25 nucleotides. Without being translated into proteins, they control gene expression and interfere with critical cellular pathways. MiRNAs have been implicated in a variety of cell functions, including cell growth, apoptosis, proliferation, and differentiation [4]. The expression of specific miRNAs which are related to bladder tumorigenesis was presented in several studies including prediction and progression [5,7,9]. MiRNAs have different expression patterns between the pathological subtypes of BC as their levels differ between the NMIBC and MIBC, suggesting that their determination is beneficial for gene and protein signatures [10]. However, interactions between miRNAs and their targeted genes are conflicting, and their physiological functions in BC cells are still unclear. This may be due to the variation in the specificity and sensitivity of the several platforms used to evaluate miRNA levels or the differences in the cohorts across studies [11].

Measuring protein expression represents a more accurate method to evaluate the activity of specific genes as it is the functional units that are derived from gene activity. Bastos et al. reported that miRNAs affected the expression of a great number of proteins, which could be a useful tool to clarify novel molecular mechanisms that promote the growth, development, and progression of BC [12]. Changes in mRNA levels induced by miRNAs indicated that the suppression of protein levels reflects transcript levels [13]. Therefore, measuring miRNA level and protein expression is necessary to understand miRNA complex activities [14].

In this study, we shed light on the expression of different miRNAs with their gene that could support the potential prognostic value in bladder cancer progression and expansion in different pathways, these pathways might be therapeutically targetable in the future which will improve the prognosis of bladder cancer. Immunohistochemistry (IHC) was selected too to study the altered proteins. We considered these to be the most practical methods to allow our findings to be easily translated into clinical practice for the management of treatment that may improve patient outcomes.

## 2. Patients and Methods

### 2.1. Patients and Sample Collection

This cohort study included 157 patients with different stages of BC who were treated at the Urology and Nephrology center. Among them, 40 of the patients were pT1 (NMIBC). The remaining 117 cases were MIBC and included 38 with pT2, 40 with pT3, and 39 with pT4. Patients with NMIBC were treated with transurethral resection (TURBT) and received intravesical treatment with BCG, while those with MIBC were treated by radical cystectomy. Samples of tissue were collected from the malignant tissue of each patient. If a patient underwent more than the TURBT procedures, we considered the first biopsy. The tissue samples were collected fresh from resection or radical cystectomy specimens then stored frozen at −80 °C for miRNA and mRNA gene expression analysis. The miRNA expression (let-7a-5p, miRNA-449a-5p, miRNA-145-3P, miRNA-124-3P, miRNA-138-5p, and miRNA-23a-5p) and target genes of miRNAs (WNT7A, β-catenin, IRS2, FZD4, SOS1, HDAC1, HDAC2, HIF1α, and PTEN) were assessed. All employed targets were enriched using an enricher online available tool, and network analysis was performed using the mirNet tool to predict direct and indirect miRNA to target gene correlation. The Institutional Review Board (IRB) of the Faculty of Medicine, Mansoura University, Egypt approved the protocol of the study (Mansoura UNC IRB #RP-17.12.301.R1).

### 2.2. Immunohistochemical Examination 

One-half of a bladder biopsy from malignant tissues was frozen for PCR, while the other half was fixed in 10% buffered formalin. Immunohistochemistry was performed on three μm-thick sections. Deparaffinized sections were incubated with hydrogen peroxide and heated in citrate buffer. Then, the immunoperoxidase approach was carried out by monoclonal antibodies of *β-catenin*, *SOS1*, *HDAC1*, *HDAC2*, *HIF1*, and *PTEN* (monoclonal mouse anti-human antibody, Santa Cruz Biotechnology, Dallas, TX, USA). Immunostaining was performed using Power-Stain™ 1.0 Poly HRP AEC Kit (Genemed Biotechnologies, San Francisco, CA, USA) with (DAB) as a chromogen. The slides were examined using an Olympus CX51 light microscope, and the diagnosis was obtained using the WHO histological classification of BC [15].

### 2.3. Real-Time PCR for Studying Gene and Target miRNA

TRIzol (Invitrogen, Waltham, MA, USA) was used to extract total RNA from serial cryosections according to the manufacturer’s protocol. Following the manufacturer’s instructions, complementary DNA (cDNA) was generated from RNA using a cDNA reverse transcription kit (Applied Biosystems, Waltham, MA, USA). MiRNAs, and miScript Reverse Transcription kit (Qiagen, Hilden, Germany) were used to reverse transcribe to cDNA.

SYBER Green PCR Master Mix (Applied Biosystems, USA) was used for mRNA QRT-PCR analysis. (*WNT7A*, *β-catenin*, *IRS2*, *FZD4*, *SOS1*, *HDAC2*, *FGFR3*, *HDAC1*, and *HIF1α*) primers with housekeeping GAPDH were obtained from Applied Biosystems, USA. Table 1 lists all the primers for the genes. qPCR assays for miRNAs were performed using the miScript SYBR-Green PCR kit (Qiagen, Hilden, Germany) and miScript primer assay for let-7a-5p, miRNA-449a-5p, miRNA-145-3P, miRNA-124-3P, miRNA-138-5p, and miRNA-23a-5p and normalized to RUN6-2 (Qiagen, Hilden, Germany).

cDNA samples and primers were utilized in StepOnePlus real-time PCR (Applied Biosystems) for quantitative PCR. The procedure includes initial denaturation for 10 min at 95 °C, followed by 40 cycles of denaturation for 15 s at 95 °C, annealing for 1 min at 60 °C, and final extension for 1 min at 72 °C. Data analysis was carried out by Equation 2^−ΔΔCT^ [16].

### 2.4. Outcome

The biological significance of the altered miRNAs and target gene expressions in BC was identified using miRDB-Base. Six miRNAs, namely, let-7a-5p, miRNA-449a-5p, miRNA-145-3P, miRNA-124-3P, miRNA-138-5p, and miRNA-23a-5p, and their nine target genes (*WNT7A*, *β-catenin*, *IRS2*, *FZD4*, *SOS1*, *HDAC1*, *HDAC2*, *HIF1α*, and *PTEN* were assessed by qRT-PCR, and a comparison between the gene expression levels was performed between pT1 and pT2–T4 stages.

### 2.5. Statistical Analysis

Quantitative data were given as means ± SD, whereas qualitative data were presented as numbers and percentages. Comparison between groups was carried out by student t-tests and correlation by Pearson’s correlation coefficient test. A strong positive correlation was considered with r values between 0.5 to 1.00 and a strong negative correlation was considered with r values between −1.00 to −0.5. For survival analysis, only patients with survival data were included (100 patients in T2–T4). The analysis was not performed for patients with T1 because of the low number. Cancer-specific survival (CSS) was defined based on the time the patient developed local recurrence and/or distant metastasis. The values of miRNA/gene expression were categorized into high/low based on a cut-off value calculated by the maximally selected rank statistics from the “*maxstat*” R package [17] (Supplementary Table S1). A Kaplan–Meier curve was constructed, and the log-rank test was used for statistical significance. SPSS version 16 (SPSS Inc., Chicago, USA) and R programming language version 3.6.3 with the “*survminer*” package were used for statistical analysis.

## 3. Results

### 3.1. Demographics

Of patients, 84.1% were males and the mean age was 57.77 with 88.53% less than 70 years. There was no significant difference between males and females regarding the median level of expression apart from miRNA23a-5p which was significantly expressed in females (*p* = 0.006). The levels of expression are displayed in Supplementary Figures S1 and S2.

### 3.2. MiRNAs and Target mRNA Gene Expression

**Figure 1:**
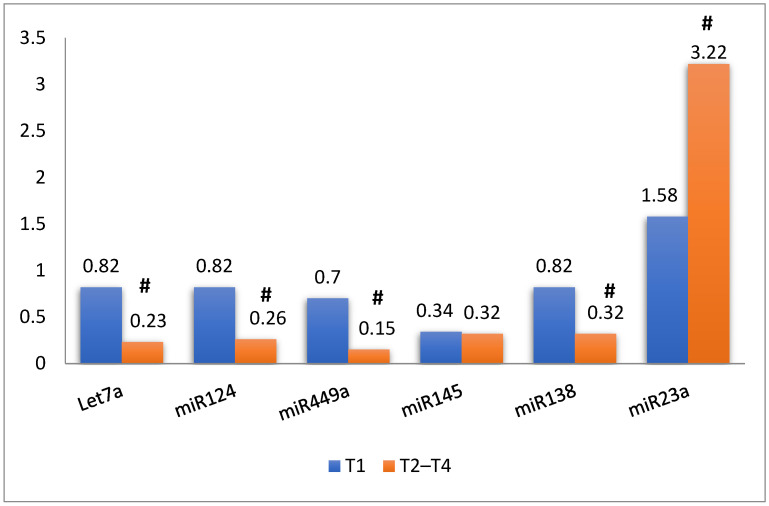
Expression of miRNAs based on BC stages. # *p* < 0.05.

A significant decrease was observed in let-7a-5p, miRNA-449a-5p, miRNA-124-3P, and miRNA-138-5p expressions in pT2–T4 compared with pT1 (*p* < 0.001). However, the miRNA-145-3p expression did not show any significant difference between stages (*p* = 0.310), while miR-23a-5p showed a significant increase in its expression with muscle invasion (*p* < 0.001) (Figure 1).

**Figure 2:**
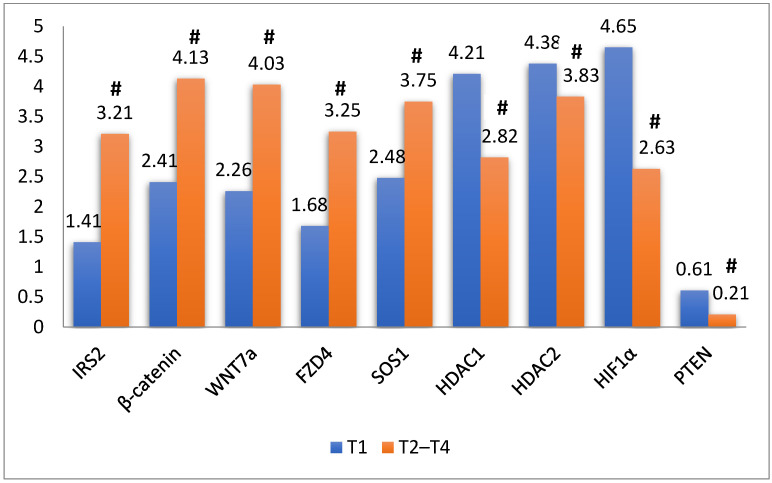
Expression of target genes on different stages of BC. # *p* < 0.05.

Let-7a-5p target genes (*WNT7A*, *β-catenin*, *IRS2*, and *FZD4*) expression significantly increased in pT2–T4 compared with pT1 (*p* < 0.001). Similarly, the *SOS1* gene targeted by miR-124-3p also showed a significant increase in its expression in pT2–T4 than pT1 (*p* < 0.001). In contrast, *HDAC2* which is targeted by miR-145-3p, *HDAC1* targeted by miR-449a-5p, *HIF1α* targeted by miR-138-5p, and *PTEN* targeted by miR-23a-5p showed a significant decrease in pT2–T4 compared to pT1 (*p* < 0.001) (Figure 2).

### 3.3. Correlation between miRNAs and Their Target Genes

The correlation between miRNAs and their target genes at pT1 and pT2–T4 stages using Pearson’s correlation coefficient was analyzed. Our results showed that miRNA let-7a-5p has a significantly strong negative correlation with only one gene (*WNT7A*) in both pT1 and pT2–T4 stages, while it showed a negative correlation with three genes (*β-catenin*, *IRS2*, and *FZD4*) in pT2–T4 stages, and low negative correlation with target gene SOS1 in pT2–T4 stages (Table 2).

### 3.4. Immunohistochemistry

**Figure 3:**
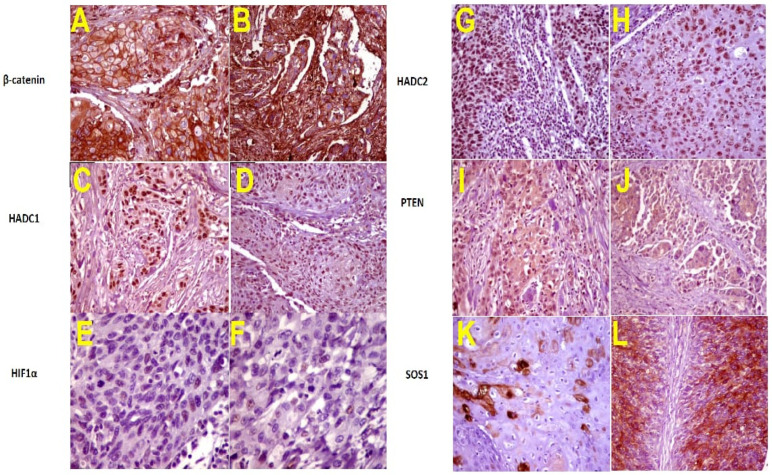
Urothelial carcinoma showing moderate positive membranous staining for *β-Catenin* in T1 stage (**A**) and positive membranous staining for *β-Catenin* in T2–T4 stage patients (**B**) (400×). HADC1 shows marked nuclear staining of tumor cells in pT1; (**C**) and moderate nuclear staining of tumor cells in T2–T4 (**D**) (200×). HIF1 showing marked nuclear staining of tumor cells in pT1 (**E**) and mild nuclear staining of tumor cells in T2–T4 (**F**) (400×), HDAC2 showing strong positive nuclear staining of surface epithelium in pT1; (**G**) and moderate positive nuclear staining of tumor cells in T2–T4 (**H**) (200×). PTEN showed moderate positive nuclear staining of tumor cells in pT1 (**I**) and mild positive nuclear staining of tumor cells in T2–T4 (**J**) (200×). SOS1 showed moderate positive nuclear staining of tumor cells in pT1 (**K**) and marked positive nuclear staining of tumor cells in T2–T4 (**L**) (200×).

No significant differences were detected between levels of *β-catenin*, *SOS1*, *HDAC1*, *HDAC2*, *HIF1*, and *PTEN* expression with age and sex of patients (*p* > 0.05). A significant difference was detected between *β-catenin* and *SOS1* expression and stage of BC. Their protein expression was significantly lower in T1 than in T2–T4 (*p* ≤ 0.05). In contrast, according to the mean percentage of positive cells, the *HDAC1*, and *HDAC2* nuclear expression was significantly higher in pT1 than in T2–T4 (*p* ≤ 0.05). Moreover, *HIF1* and *PTEN* showed lower expression in T2–T4 than pT1 (*p* ≤ 0.05) as presented in Table 3 and Figure 3A–L. Immunohistochemical staining data and the clinicopathological variables and their correlation with protein expression are summarized in Table 3.

### 3.5. Prognostic Effect on CSS

**Figure 4:**
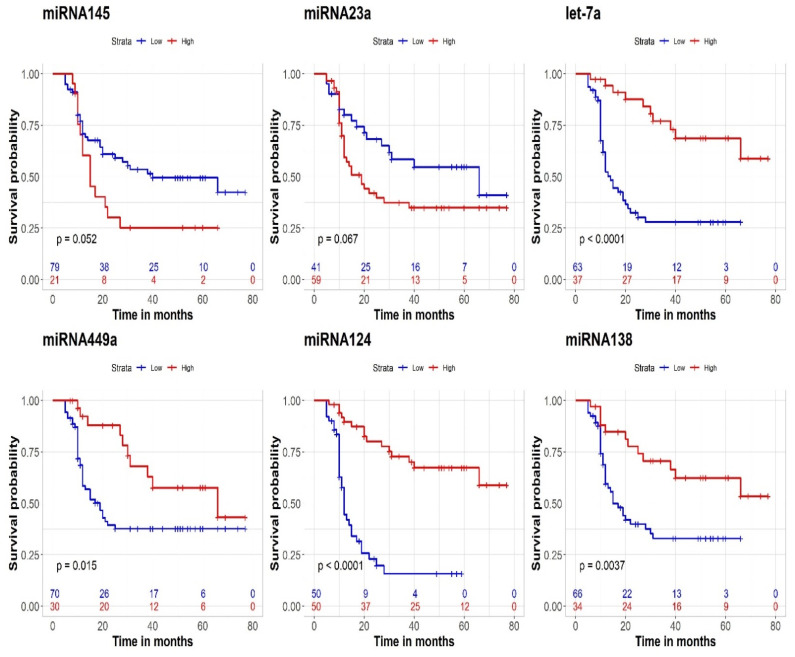
Prognostic effect of miRNAs; let-7a-5p, miRNA-449a-5p, miRNA-145-3P, miRNA-124-3P, miRNA-138-5p, and miRNA-23a-5p on CSS.

**Figure 5:**
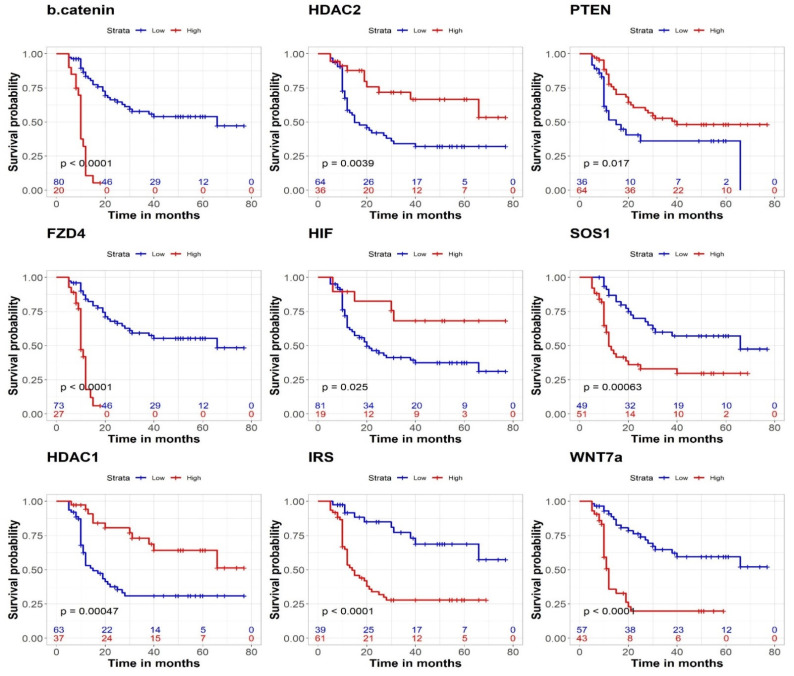
Prognostic effect of targeted genes; *β-Catenin, WNT7A, IRS, FZD4, SOS1, HDAC1, HDAC2, HIF1α*, and *PTEN* on CSS.

Lower CSS was significantly related to lower expression of let-7a-5p (*p* < 0.001), miRNA-124-3p (*p* = 0.01), miRNA-499a-5p (*p* < 0.001), and miRNA-138-5p (*p* < 0.001). Conversely, the expression levels of miRNA-145-5p and miRNA-23a-5p did not reach statistical significance (Figure 4). For gene products, higher expression of *β-catenin*, *FZD4*, *IRS2*, *WNT7a*, and *SOS1* was significantly associated with worse CSS. In contrast, lower levels of *HDAC1*, *HDAC2*, *HIF1α*, and *PTEN* were associated with lower CSS (Figure 5). The values of 1- and 5-year CSS are demonstrated in Table 4.

## 4. Discussion

MicroRNAs are directly involved in many vital processes, including cell proliferation, differentiation, apoptosis, migration, and metabolism, which are all cancer-related pathways. Studies have revealed the potential prognostic role of microRNAs in BC and that miRNAs are associated with progression and survival in patients initially presenting with disease [5,11,18,19].

In the current study, we identified six expressed miRNAs: four downregulated in MIBC, one upregulated in MIBC (miR-23a), and one showed no statistical difference between NMIBC and MIBC (miR-145). Most of these miRNAs were previously reported in regulating BC malignancy [20]. Several studies have established a link between miRNA let-7a expression and BC progression and/or survival [21]. Our results illustrated that let-7a-5p has a reverse correlation with the stage, its expression was downregulated in pT2–pT4 compared to pT1, in agreement with [22] who showed decreased let-7a expression was associated with pT2–pT4 with worse survival. On the other hand, miR-124 was reported as a tumor suppressor in the prostate and BC [23,24]. This finding agreed with our results that showed downregulation of miR-124-3p in high stages pT2–pT4.

Recent studies have indicated the downregulation of miR-449a in several malignant tumors [25,26]. Chen et al. [10] showed a decrease in miR-449 expression in the tumor tissue of BC patients and cell line. This outcome was parallel with our result that showed a significant reduction in miR-449-5p in pT2–pT4 compared to pT1.

Previous research has suggested that miR-145 suppresses tumors in humans through reducing the expression of oncogenic genes. MiR-145 manifested a lower level of expression in BC tissue compared to the normal control [27]. MiR-145 was downregulated in BC, but its expression with the histological grading and staging was not correlated [28]. Our data showed no statistical difference in miR-145-3p expression between different stages.

Earlier studies mentioned miR-138 as a tumor suppressor in human cancers, and upregulation of miR-138 was found to suppress the proliferation and invasion of cancer cells [29]. Our study supported this hypothesis and showed a significant decrease in miR-138-5p in the high stages of BC. MicroRNA-23a, which acts as an oncogene, was reported to be increased in renal cell carcinoma cell lines and associated with lower survival in renal cancer patients [30]. In our study, miRNA-23a-5p significantly increased with high stages BC.

With the application of negatively correlated coefficients in miRNA–mRNA identification, miRNA target gene pairs have been proposed to play a role in the progression of tumors [31]. Wnt/β-Catenin is implicated in cancer biology via several mechanisms. Wnts bind to members of seven transmembrane families to stimulate complex network events dependent on β-catenin (CTNNB1) (canonical pathway) or independent of β-catenin (non-canonical pathways) [32]. In addition, Wnt pathway alterations were detected in many kinds of cancer, confirming its importance during bladder carcinogenesis [33]; a finding compatible with our results that showed a significant elevation in WNT7A and CTNNB1 expression in high stages.

Low expression of let-7a in liver cancer cells because of its inhibitory effect on the Wnt signaling pathway by regulating Frizzled Class Receptor 4 (*FZD4*) gene at megakaryocyte development was indicated [34]. These findings confirm our results that downregulation of let-7a-5p expression was related to increasing FZD4 and CTNNB1 expression in high stages of BC. Through our results, let-7a-5p exhibited a strong negative correlation with its target gene WNT7A at low and high stages, and with CTNNB1and FZD4 at high stages of pT2–pT4.

In addition, it has been shown that *IRS* could be a potential diagnostic marker for urothelial carcinomas of the bladder in humans [35]. Our results revealed an upregulation of *IRS2* expression in high stages of pT2–pT4 compared to low stage pT1. Furthermore, there was a strong negative correlation between let-7a-5p and its target gene IRS. IRS1/2 overexpression elevated the dishevelled2 (Dvl2) protein level and promoted Wnt/β-catenin signaling, as demonstrated by the increase in T cell-specific factor 4, CYCLIN D1, and c-MYC. IRS1/2 overexpression stimulated metastasis and tumor formation. Likewise, IRS1 downregulation by miRNA-145 inhibited tumor metastasis [35].

Son of sevenless homolog 1 (*SOS1*) gene, which is a stimulator of Ras/MAPK in intrauterine development, was found to be upregulated in bladder and prostate cancer by ERK signaling activation, which is linked to higher stages of cancer and is a factor for cancer aggressiveness [36]. This information agrees with our findings highlighting a significant increase in *SOS1* expression with higher stages. On the other hand, miR-124-3p was stated as a suppressor of tumors in prostate and bladder cancer and was identified as a target of the *SOS1* gene [23,24].

Histone deacetylases (*HDACs*) have a major role in regulating transcription and in cellular processes. *HDAC-1* and *HDAC-2* were found to be strongly related to pT1 high-grade papillary bladder tumors, and their high levels of expression were linked to a shorter progression-free survival [37]. These findings are in harmony with our results that showed a reduction in *HDAC1* and *HDAC2* genes level with high stages. MiR-449a, which has a growth suppressing activity thought to have an inhibitory activity on *HDAC-1* expression, has been confirmed with the reduction in its levels with high stages [38]. Noonan et al. reported that miR-145 suppresses *HDAC2* overexpression in hepatic cell carcinoma tumorigenesis [37]. On the other hand, miR145 was not significantly correlated with its target gene HDAC2 in T1 or T2–T4 diseases in our samples. This controversy might be because of our sample size and possibly other regulatory factors that affect gene expression but have not been studied. HDAC1 and HDAC2 are essential components of the transcriptional complexes SIN3A, NuRD, and CoREST, which are recruited to promoters by DNA binding proteins to induce gene-specific transcriptional suppression. HDAC1 and HDAC2 restrict the production of p21WAF1/CIP1 and p57KIP2, regulating the cell cycle’s transition from G1 to S. Interestingly, NLK serine/threonine kinase phosphorylation of HDAC1 at the critical serine 421 results in reduced β-Catenin/LEF1 promoter, indicating the ability of the NLK-HDAC1 axis to downregulate WNT signaling, which is critical for the prevention of aberrant proliferation of non-transformed, primary fibroblast cells. Similarly, the c-ABL (ABL1/ABL) protein kinase can stimulate tyrosine phosphorylation of HDAC2, which is the primary function of the oncogenic fusion protein BCR-ABL [8].

Blood vessel formation is essential in cancer development and progression. The correlation between the density of tumor microvascular architecture and tumor stage was described in many kinds of cancers [39,40]. Hypoxia-Inducible Factor (*HIF-1*) is activated by the hypoxic conditions in tumor development in the angiogenesis phase. Recently, it was pointed out that the *HIF-1* pathway contributes highly to tumor growth and progression. Nevertheless, the relation between tumor stage and *HIF1α* in BC is unclear [41,42]. Our study showed an inverse relationship between *HIF1α* expression and tumor stage, with significantly lower *HIF1α* levels in high stages of pT2–pT4, a finding in agreement with [43]. Furthermore, miR-138 was found to suppress the proliferation and invasion of cancer cells by inhibiting *HIF1α* [29].

Phosphatase and tensin homolog (*PTEN*) is one of the most frequently deleted or mutated genes in tumors. Its functions in converting phosphatidylinositol-3, 4, 5-triphosphate (PIP3) into a diphosphate product (PIP2) slow tumor progression and prevent the development of many cancers [44]. Our results showed the downregulation of *PTEN* in high stages.

Besides the direct action of miRNAs on the target genes, miRNAs may be suppressed at the translational and protein transduction level [45] and can alter the expression levels of target proteins [46]. Our immunohistochemical study revealed six expressed proteins were interconnected, suggesting that miRNAs may regulate proteins in the same manner indicated by the mRNA expression profile.

The overexpression of *CTNNB1* and *SOS1* proteins was observed in higher stages (T2–T4). Some studies, in line with ours, recommended the use of *CTNNB1* as a marker of tumor progression [47,48]. In contrast, *HIF1* and *PTEN* showed marked expression in low-stage pT1, which was confirmed by the correlation between miRNA and mRNA. Moreover, *HDAC1* and *HDAC1* revealed lower protein expression in pT2–pT4 compared to low stage pT1, a finding compatible with Poyet et al. who found that *HDAC1* and *HDAC* 2 have a rough prognostic relevance in pTa and pT1 tumors [37].

Let-7a target gene (*WNT7A*, *β-catenin*, *IRS2*, and *FZD4*) expression significantly increased in T2–T4 compared with pT1 as let-7a miRNA inhibition stimulates *FZD4* and *Wnt/β-catenin* pathway in cancer cells. Similarly, the *SOS1* gene targeted by miR-124 also showed a significant increase in its expression in pT2–T4 compared to pT1. In contrast, *HDAC2* which is targeted by miR-145, *HDAC1* targeted by miR-449a, *HIF1α* targeted by miR-138, and *PTEN* targeted by miR-23a, showed a significant decrease in pT2–T4 compared to pT1.

## 5. Conclusions

These results suggested that the link between let-7a-5p, miRNA124-3p, miRNA138-5p, and their target genes could be a promising tool in the prognosis of BC. The critical role of miRNA–mRNA correlations would be helpful to find specific biomarkers for BC prognosis.

## Figures and Tables

**Table 1:** Primer sequences used for quantitative RT-PCR.

Common Name	Sequence	Accession No.
*WNT7a*	F-5′-ACTTAGGGGTAAGGAGGGGC-3′ R-5′-GCTGGACCCAAAGCAAAGTG-3′	NM_004625.4
*β-catenin*	F-5′-CTGAGGAGCAGCTTCAGTCC-3′ R-5′-ATTGCACGTGTGGCAAGTTC-3′	NM_001098209.2
*IRS2*	F-5′-CCACTGACAACGAGAGCCAT-3′ R-5′-CCCATGGCCCCAGTGTTTAT-3′	NM_005544.2
*FZD4*	F-5′-CCAACTGGGCACTTTTTCGG-3′ R-5′-TCTAAACAGCAGACAGCGCA-3′	NM_012193.4
*SOS1*	F-5′-TGTGCAAGGCCATGGATACC-3′ R-5′-CCTTGTCAGCACACATTGCC-3′	NM_001382394.1
*HDAC1*	F-5′-ACTGCTAAAGTATCACCAGAGGG-3′ R-5′-CACACTTGGCGTGTCCTTTG-3′	NM_004964.3
*HDAC2*	F-5′-GAGGTGGCTACACAATCCGT-3′ R-5′-TAGCCACTGAAACAAGACTTCA-3′	NM_001527.4
*HIF1α*	F-5′-ACTTGGCAACCTTGGATTGGA-3′ R-5′-GCACCAAGCAGGTCATAGGT-3′	NM_001243084.1
*PTEN*	F-5′-AGCTGGAAAGGGACGAACTG-3′ R-5′-ACACACAGGTAACGGCTGAG-3′	NM_001304717.5
*GAPDH*	F-5′-GAAGGTGAAGGTCGTAGTC-3′ R-5′-GAAGATGGTGATGGGATTTC-3′	NM_001357943.2

**Table 2:** The correlation between miRNAs and targeted genes at T1 and T2–T4 stages.

miRNA	miRNA Regulated Status	Target Gene	Gene Regulation Status	Pearson’s r (*p*-Value)	
				T1	T2–T4
Let-7a-5p	down	*WNT7A*	up	−0.530 (*p* < 0.001)	−0.687 (*p* < 0.001)
Let-7a-5p	down	*β-catenin*	up	0.418 (*p* = 0.007)	−0.687 (*p* < 0.001)
Let-7a-5p	down	*IRS2*	up	0.374 (*p* = 0.018)	−0.664 (*p* < 0.001)
Let-7a-5p	down	*FZD4*	up	−0.323 (*p* = 0.042)	−0.733 (*p* < 0.001)
miR124-3p	down	*SOS1*	up	−0.630 (*p* < 0.001)	−0.579 (*p* < 0.001)
miR449a-5p	down	*HDAC1*	up	−0.326 (*p* = 0.04)	0.369 (*p* < 0.001)
miR145-3p	down	*HDAC2*	up	0.271 (*p* = 0.091)	−0.123 (*p* = 0.186)
miR138-5p	down	*HIF1α*	up	−0.601 (*p* < 0.001)	0.510 (*p* < 0.001)
miR23a-5p	up	*PTEN*	down	0.660 (*p* < 0.001)	−0.513 (*p* < 0.001)

**Table 3:** Correlation between miRNAs target protein expressions with the clinicopathological characteristics of bladder cancer patients.

		Gender			Age			Tumor Stage		
		Male	Female	*p*-Value	<70 Years	≥70 Years	*p*-Value	T1	T2–T4	*p*-Value
*β-Catenin*	High	96 (72.72%)	17 (68%)	0.464	86 (61.87%)	11 (61.11%)	0.239	9 (22.5%)	88 (75.21%)	0.003
	Low	36 (25.89%)	8 (32%)		53 (38.12%)	7 (38.88%)		31 (77.5%)	29 (24.78)	
*HDAC1*	High	72 (58.5%)	11 (44%)	0.182	77 (57.5%)	6 (42.9%)	0.295	34 (85%)	45 (38.46%)	0.001
	Low	51 (41.5%)	14 (56%)		57 (42.5%)	8 (57.1%)		6 (15%)	72 (61.54%)	
*HIF1α*	High	50 (37.9%)	7 (28%)	0.346	54 (40.3%)	3 (21.4%)	0.167	29 (72.5%)	28 (23.9%)	0.000
	Low	82 (62.1%)	18 (72%)		80 (59.7%)	11 (78.6%)		11 (27.5%)	89 (76.1%)	
*SOS1*	High	81 (61.4%)	20 (80%)	0.074	94 (70.1%)	7 (50%)	0.123	16 (40%)	85 (72.6%)	0.000
	Low	51 (38.6%)	5 (20%)		40 (29.9%)	7 (50%)		24 (60%)	32 (27.4%)	
*PTEN*	High	46 (34.8%)	7 (28%)	0.507	50 (37.3%)	3 (21.4%)	0.238	28 (70%)	25 (21.4%)	0.000
	Low	86 (65.2%)	18 (72%)		84 (62.7%)	11 (78.6%)		12 (30%)	92 (78.6%)	
*HDAC2*	High	64 (48.5%)	9 (36%)	0.251	67 (50%)	67 (50%)	0.309	32 (80%)	41 (35.04%)	0.000
	Low	68 (51.5%)	16 (64%)		5 (35.7%)	9 (64.3%)		8 (20%)	76 (64.95%)	

**Table 4:** 1- and 5-yr cancer-specific survival (CSS) for miRNA and gene products expression.

Expression		1-Year CSS (%)	5-Year CSS (%)	*p*-Value
miRNA				
Let-7a-5p	Low	61.9	27.9	<0.001
	High	94.3	68.9	
miRNA-449a-5p	Low	57.7	15.7	<0.001
	High	89.5	67.2	
miRNA-145-3P	Low	77	49.5	0.05
	High	70.4	25	
miRNA-124-3P	Low	68.5	37.6	0.01
	High	92.3	57.7	
miRNA-138-5p	Low	68.7	32.8	0.004
	High	84.7	62.3	
miRNA-23a-5p	Low	80	54.7	0.06
	High	69.9	34.8	
Gene products				
*β-catenin*	Low	86.5	54	<0.001
	High	32	-	
*FZD*	Low	87	55	<0.001
	High	41.9	-	
*HDAC1*	Low	62.4	31	<0.001
	High	94	64	
*HDAC2*	Low	67.6	32	0.004
	High	87	66.6	
*HIF*	Low	71.9	37.4	0.02
	High	89.5	68	
*IRS*	Low	91.5	68.8	<0.001
	High	64.8	27.9	
*PTEN*	Low	58.3	36	0.01
	High	85	48	
*SOS-1*	Low	93.5	57	0.01
	High	59.8	29.7	
*WNT7a*	Low	92.9	59.4	<0.001
	High	50.7	19.7	

Cut-off values were determined based on the maximally selected rank statistics method.

## Data Availability

All data generated during this study are included in this published article (and its supplementary information files).

## Supplementary Materials

The following supporting information can be downloaded at: https://www.mdpi.com/article/10.3390/genes13091629/s1, Figure S1: Prognostic effect of miRNAs; let-7a-5p, miRNA-449a-5p, miRNA-145-3P, miRNA-124-3P, miRNA-138-5p, and miRNA-23a-5p on CSS in males and females; Figure S2: Prognostic effect of targeted genes; *β-Catenin*, *WNT7A*, *IRS*, *FZD4*, *SOS1*, *HDAC1*, *HDAC2*, *HIF1α*, and *PTEN* on CSS in males and females; Table S1: the cut-off values used to categorize the level of expression into low/high.
