# Supplementary material for: Identification of Epigenetic Interactions between miRNA and Gene Expression as Potential Prognostic Markers in Bladder Cancer
Source: Genes (Basel). 2022 Sep 10;13(9):1629. doi: 10.3390/genes13091629 (PMC9498328; doi:10.3390/genes13091629)
Supplement: Supplementary file 1 [file genes-13-01629-s001.zip › genes-1880656-supplementary.pdf]

**Supplementary Table S1: the cut-off values used to categorize the level of expression into low/high**

| miRNA/Gene product | Cut-off |
|---|---|
| Let-7a-5p | 0.24 |
| miRNA-449a-5p | 0.18 |
| miRNA-145-3P | 0.5 |
| miRNA-124-3P | 0.19 |
| miRNA-138-5p | 0.4 |
| miRNA-23a-5p | 2.9 |
| β-catenin | 4.65 |
| FZD4 | 3.54 |
| HDAC1 | 2.98 |
| HDAC2 | 4.08 |
| HIF | 3.01 |
| IRS | 2.98 |
| PTEN | 0.15 |
| SOS-1 | 3.598 |
| WNT7a | 3.98 |

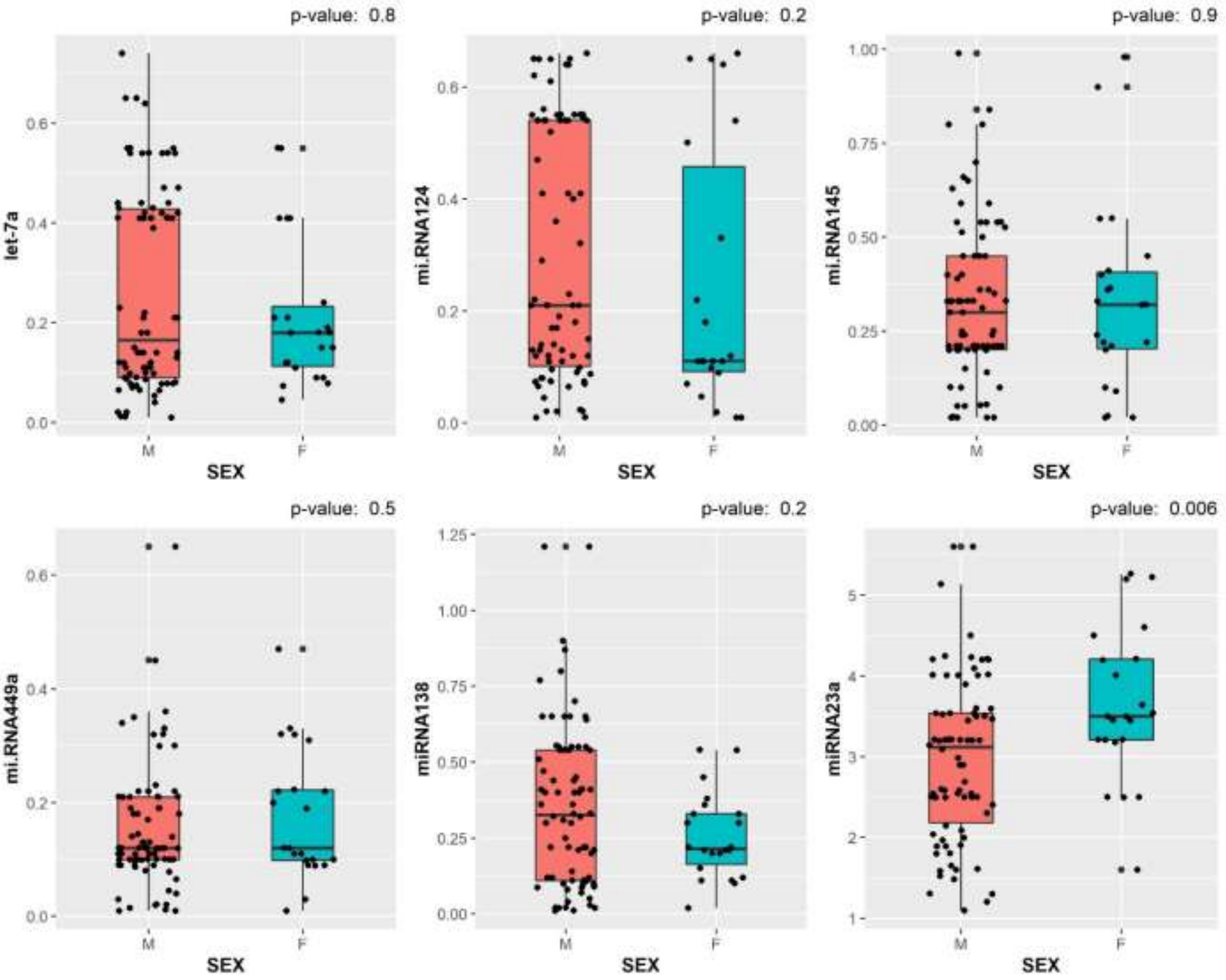


**Supplementary Figure S1:** Prognostic effect of miRNAs; let-7a-5p, miRNA-449a-5p, miRNA-145-3P, miRNA-124-3P, miRNA-138-5p, and miRNA-23a-5p on CSS in males and females.

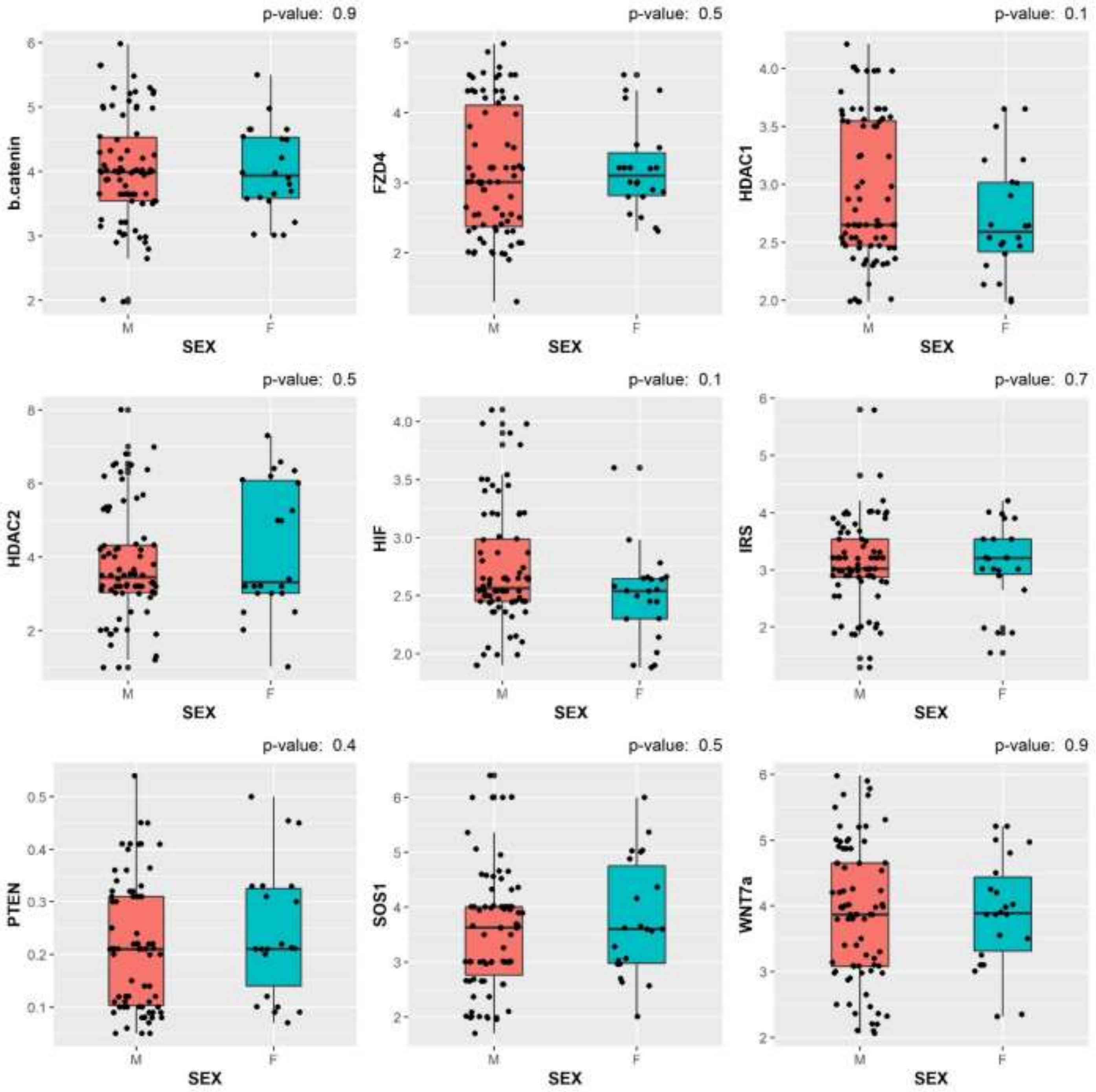


**Supplementary Figure S2:** Prognostic effect of targeted genes; β-Catenin, WNT7A, IRS, FZD4, SOS1, HDAC1, HDAC2, HIF1$\alpha$, and PTEN on CSS in males and females.
